# Noise Generation and Acoustic Impact of Free Surface Hydropower Machines: Focus on Water Wheels and Emerging Challenges

**DOI:** 10.3390/ijerph182413051

**Published:** 2021-12-10

**Authors:** Emanuele Quaranta, Gerald Müller

**Affiliations:** 1 European Commission Joint Research Centre, 10127 Ispra, Italy; 2 Engineering and Physical Sciences, University of Southampton, Southampton SO17 1BJ, UK; g.muller@soton.ac.uk

**Keywords:** acoustic impact, low head hydropower, micro hydropower, noise, water wheel

## Abstract

The noise generated by free surface hydropower machines, e.g., water wheels, has led to complaints and to restrictions in their operation in urban areas. This problem generally occurs when water wheels are not well designed and are installed without expertise. Despite the relevance of the problem, and the growing interest in the use of water wheels at existing low head barriers, the acoustic impact of water wheels has not yet been properly addressed by the scientific community. Therefore, in this manuscript, the importance of the problem and the related scientific challenges are discussed, supported by case studies and theoretical considerations. A literature review on the topic is carried out, although little information is available in the scientific domain. The aim of this work is to increase the awareness on this problem, in order to stimulate future research and to suggest useful guidelines for future water wheel projects, thereby increasing the water wheel potential and reducing noise disturbance for people.

## 1. Introduction

Gravity hydraulic machines use water weight to generate energy. The two most known types of gravity machines are gravity water wheels, used in the past to drive water mills [1], and Archimedes screws [2]. Gravity water wheels are the focus of this study.

Gravity water wheels are cost-effective hydropower converters for the repowering of old mill sites and for the retrofitting of non-powered low head weirs (typically below 2.5 m) and small flows (below few cubic meters per second). Their advantages are flexible operation at off-design conditions, high peak hydraulic efficiency (up to 85%), and low costs in comparison to analogous reaction turbines, e.g., low head Francis and Kaplan turbines [3]. Furthermore, water wheels operate with a fish-friendly behavior and are compatible with sediment [3,4]. The most recent review papers on gravity water wheels have been published in [5,6], where their engineering design, operation, and historic development have been discussed.

Gravity wheels can be classified into three main types, depending on their inflow configuration.

**Figure 1:**
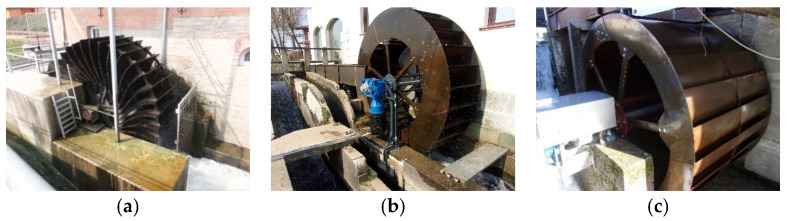
(**a**) Undershot water wheel (Zuppinger type), picture of G. Müller; (**b**) middle breastshot wheel (photo courtesy of Gatta srl), The power take off and the blue gearbox.; and (**c**) overshot wheel (photo courtesy of Gatta srl).

Undershot wheels, typically used below 1.5 m head and maximum flow below 1000 L/s per meter width, with a maximum hydraulic efficiency of 80% and global plant efficiency of 65% [7], depending on the head and flow rate; the inflow is located in the lowest portion of the wheel (Figure 1a). They can be of two main types: Sagebien type with flat blades and Zuppinger type with curved blades [7].

Breastshot wheels, typically used below 3 m head and with a maximum flow below 600–800 L/s per meter width, with a maximum hydraulic efficiency of 80% and global plant efficiency of 65% (Figure 1b). They can be classified as low breastshot (i.e., undershot), middle breastshot (the water enters near the rotation axis) and high breastshot (the water enters in the upper third of the wheel) [8,9].

Overshot wheels, typically used between 3 m and 6 m head and with maximum flows below 200 L/s per meter width, with a maximum hydraulic efficiency of 85% and global plant efficiency of 70% [10]. The inflow is at the top of the wheel, and they are usually installed in mountain areas (Figure 1c).

Data collected during the Restor Hydro project, described in detail in [11], showed that the repowering of EU (European Union) historic sites with low heads weirs and mills would produce 8703 TWh/y. France, with the potential generation of 3832 GWh/y, is the leader. In addition, 6.8 TWh/y of electricity can be generated at these historic sites by improving the infrastructures; 27,749 mills were registered and 17,485 were found to be in good status, with weirs that could be retrofitted quite easily. It was found that the majority (90%) of registered water mill sites had a capacity lower than 40 kW and could be possible sites for water wheel installations. The potential is not only limited to EU. For example, in an irrigation canal system in Pakistan (Khyber Pakhtunkhwar/Peshawar), there are 1267 drop structures with head differences between 0.5 and 3 m, power ratings between 2 and 100 kW, with a total hydraulic power of 68.6 MW over a canal length of 1120 km (pers. comm. Gerald Müller). In most of these sites, water wheels could be installed as cost-effective hydropower converters. Pakistan has a total length of 40,000 km of primary canals, so that the actual potential is much larger.

However, water wheels also exhibit some drawbacks, e.g., low rotational speeds (i.e., expensive gearboxes), large diameters and, sometimes, and noise emission during operation. Noise emission is generally associated with the inflow phenomena and the water uplift at the outflow.

Nevertheless, the acoustic impact of water wheels has been poorly investigated by the scientific community, and only few data and case studies are available in the literature. The only available review paper deals with the noise generated by hydraulic weirs for low head applications [12]. Therefore, the scope of the present opinion paper is to discuss the available data and publications on the noise generated by water wheels. The scientific gaps and challenges to be addressed are highlighted, and some guidelines to reduce the noise of water wheels are discussed. The available case studies are described.

## 2. Noise Generation from Water Wheels

Noise is defined as an unwanted and unpleasant sound, which is loud or disruptive to hearing. Noise is generated by vibrations through a medium, such as air or water [13]. Humans can hear sound frequencies higher than 15 to 20 Hz, so these signals are in the infrasonic range.

The main influences of ultra-low frequency sound on humans are thought to be the resonance of body cavities, and the effect of resonance and standing sound waves in enclosed spaces such as rooms [14]. In the former case, air or fluid filled cavities of the body can respond dynamically to infrasound, which can lead to resonance and a build-up of amplitude. These phenomena are worsened in urban areas [15,16]. In [17], it is reported that the installation of a large fan in a workshop led to a feeling of discomfort and to visual disturbances (“apparitions”) experienced by the workers. This was traced back to the fact that the fan created infrasound waves, which led to a resonance in the eyeballs of workers (the estimated resonance frequency according to NASA is 18 Hz), which in turn caused the discomfort. In the latter case, the resonance of air-filled enclosed spaces is known as Helmholtz resonance. During dynamic excitation, the air inside a containment that has one opening starts to vibrate. In the car industry, this is termed “car window buffeting”, and is known to create a feeling of discomfort [18]. The resonance amplitude here can be significantly higher than the amplitude of the exciting signal. The Helmholtz-type response is a harmonic oscillation or a standing wave that, contrary to the pulsed signal from, e.g., a water wheel, may not be audible. This is still a topic of ongoing scientific discussion (see, e.g., the reviews in [19,20]).

Due to the free water surface operation, the generated noise from water wheels may be well audible to people, so that it can become a major drawback in some cases. The noise generation in water wheels usually occurs through either the sudden contact of a blade with the surface of the inflowing water, generating a slapping noise, or through turbulence and water being lifted up and falling down on the downstream side. In both cases, it is not a harmonic sound wave, but a pulsed noise signal with pulse frequencies of 2 to approximately 10 Hz, depending on the wheel speed and blade number.

**Figure 2:**
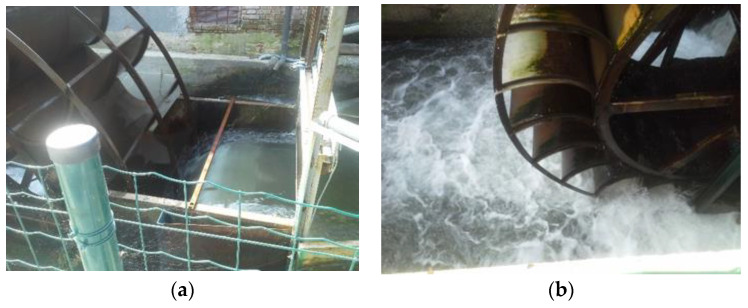
(**a**) Water wheel with bad inflow design and blade shape, and the outflow design at the tailrace (**b**) (personal collection of the author).

The problem of noise is worsened when the hydraulic design is overlooked (Figure 2). Indeed, water wheels are generally considered simple machines rather than full-fledged hydraulic turbines; therefore, they are usually manufactured by carpentry companies or blacksmiths with not enough competence on hydraulics and on water wheel design. The impact of the blades on the water jet, and possibly the noise generated when a blade exits the tailrace can generate pulsed noise signals. In addition, the generator and the gearbox can be the cause of higher frequency noise. All of these sources can result in a noise emission that is more disturbing than that generated by the free waterfall in undisturbed conditions. The noise of the generator, especially when the electro-mechanical equipment is not inside a proper powerhouse, may also worsen the acoustic impact. Noise emitted by the gearbox is omitted here, as this noise usually indicates that the component is starting to fail.

Some examples of the noise generated by water wheels can be found in the literature (Table 1). An overshot wheel in Pader (Germany) suspended its operation due to the neighboring residents’ complaints about the pulsating noise. Bristle elements were installed in the paddles to reduce the noise. A Zuppinger water wheel in Germany had blades that slammed on the upstream free surface, generating a pulsating noise [5].

Noise emission due to a wheel blade striking the water surface was also found during laboratory tests of Zuppinger water wheels, but it did not occur for Sagebien water wheels, due to the optimal blade inclination at the inflow [7]. In Italy, some examples have also been recorded by one of the authors, where the owner of the plant was obliged to stop the wheel operation after complaints from the neighbors, with serious economic consequences for the owner. An additional Italian example is that of a water wheel 6 m in diameter and 2 m wide, operating under 2 m head and 1.25 m^3^/s of flow. The noise emission from the wheel was 9 dB higher than the noise without the wheel (caused by the free waterfall). The installation of acoustic barriers reduced the noise level by 7 dB. However, the dB value is not necessarily a measure of the effect of this noise.

From a Zuppinger wheel near Freiburg, Germany, the frequency of the pulsating noise was estimated as 2–3.5 Hz, assuming a diameter of 7 m, 48 blades, and a maximum tangential velocity of the blade of 1.5 m/s. One particular feature of the pulsed noise in this case was the resonance of air filled cavities such as rooms or spaces between walls, which could amplify the signal significantly, and cause feelings of discomfort [14]. It must be noted that the acoustic pressure caused by resonance can be significantly higher than that of the arriving acoustic signal. The fact that air is resonating in a harmonic oscillation can mean that, despite the fact that the pulsating original signal is audible (as a series of noise signals similar to, e.g., a motorbike engine), the response of the air can be below the human hearing limit.

There is also a supercritical flow wheel in a school in Munich (St. Anna-Gymnasium) that can only run during working hours because of noise generation [21].

Nevertheless, it must be noted that a lot of water wheels are currently in operation with no substantial acoustic impact [5,6].

## 3. Assessment of Noise Emissions from Water Wheels

**Figure 3:**
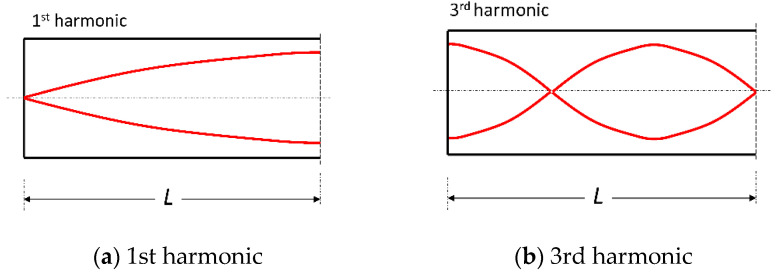
Standing acoustic wave in an enclosed space.

The noise generated by water wheels is mostly a pulsed noise rather than a harmonic oscillation. This means that, when using standard measurement devices, its amplitude is averaged and thereby reduced. Instead of measuring an average value, a time series of the noise should be taken and analyzed. Furthermore, the effect of these infrasound signals needs to be evaluated, keeping possible resonance phenomenon in mind. The sound pressure should be measured not just in the open spaces, but also in enclosed spaces (e.g., rooms with a window slightly open towards the source, or wherever complaints have been made), or even between walls. The sound pressure can locally be significantly higher than the pressure signal from the water wheel. Care should be taken to allow for the fact that the resonant waves are standing waves, so that they will have node points where the effect disappears and crest points where a maximum occurs. Figure 3 indicates the problem, while for a first harmonic wave the maximum occurs at the opening, the third harmonic has a maximum at the wall. The wrong choice of the measurement point at a node may result in a zero reading.

It appears that low-frequency signals can travel further than high-frequency noise. In addition, low-frequency noise can propagate between reflective boundaries such as walls on both sides of a street. This can increase their effective propagation distance significantly.

## 4. Outlook and Challenges

Despite the importance of the topic, the abovementioned examples are the limited data that have been found in the literature for water wheels, with low possibility of any generalization or scientific elaboration. In general, the studies investigating the acoustics of any type of micro-hydro turbine operating at atmospheric pressure are very limited [21]. The most up-to-date scientific paper on noise emission in micro hydropower plants is [12], where a comprehensive field study to evaluate the sound environments around a selection of typical low head weir sites was presented, but the study is limited to the noise emission of waterfalls from weirs. Therefore, the absence of noise-related data of water wheels is a great scientific gap that need to be addressed, especially because the assessments of noise levels are generally mandatory in sensitive areas (e.g., urban areas) before installing a hydropower plant. Neighbors, and related activities, have been noted one of the most noise annoying sources [22]. Furthermore, it can be expected that the noise intensity is correlated with the efficiency of the wheel. The better the design, the lower the noise emission of the wheel, although it cannot be reduced to zero.

The general design guidelines that should be considered to minimize noise emissions are:

(1) Respecting the triangle velocity theory at the inflow to minimize shock losses of the water flow on the blades of the wheel and reducing power losses [23,24].
(2) Minimizing water uplift downstream by choosing an optimal blade shape and a suitable diameter, reducing the noise generated by the splashing water uplift by the blade and falling down onto the tailrace. This requires the blade profile to be perpendicular to the free surface downstream [7].
(3) Enclosing the generator in a proper powerhouse.
(4) Installing artificial acoustic barriers [25] or natural (vegetation) barriers [26,27].

In all water wheel projects where noise problems may occur, it is also recommended to record the existing noise levels and characteristics at various points to create a solid database for comparison with post-implementation noise.

There is, however, a lot of research required. The fact that water wheel noise mostly consists of regular pulsed signals that can create acoustic resonance is not considered currently at all. The following research gaps should be filled:

(1) The noise emission of a water wheel under different operating conditions, i.e., at the design flow and at part and full load, need to be measured and analyzed to establish its typical characteristics
(2) Is there a correlation between noise emission and wheel efficiency?
(3) The damping of pulsating low-frequency noise, and the effective propagation distance of such noise signals, needs to be investigated, low-frequency and pulsed noise appears to travel further than high frequency noise.
(4) Potential resonance effects in enclosed air spaces should be analyzed, which could be rooms or outdoor areas enclosed by large buildings.
(5) Low frequency sound is more difficult to suppress than high frequency signals. Methods to reduce sound emissions need to be identified.
(6) The potential health effects of low-frequency or infrasound need to be investigated further.

The importance of the topic is relevant, and it is of interest also for the other turbines operating at atmospheric pressure, e.g., wind turbines [28,29] and Archimedes screws [15,16]. A study on the possibility of noise reduction on Archimedes screws was reported by [15]. The study was initiated as an Archimedes screw built in Munich had to stop operation after nearby residents complained about the noise. In this study, however, only the noise level was reported and not the frequency. A modification of the exit geometry of the screw led to a noise reduction of 12 dB, with an original noise level in the laboratory tests of 40 dB.

## 5. Conclusions

A collection of case studies and related scientific studies show that the problem of noise generation by water wheels should not be overlooked. The noise emission strictly depends on the water wheel design, with typical pulsed signals with frequencies between 2 and 10 Hz, i.e., in the infrasonic range. Such signals can lead to acoustic resonance in enclosed air spaces, where the signal amplitude is amplified significantly. This in turn can have effects on the well-being of people, especially in residential neighborhoods, and serious consequences for the operation of hydropower installation.

However, there is very little knowledge about this aspect of water wheel design. Therefore, more efforts should be spent by both the industry to develop more efficient water wheels, and by academia to study the problem of noise and to provide engineering tools to be used in practical applications. The relevance is increased by its importance also in other similar energy contexts, e.g., for wind turbines and Archimedes screws.

## Figures and Tables

**Table 1:** Case studies found in the literature.

Type	Country	Dimensions	Acoustic Impact
overshot	Germany		
undershot	Italy	2 m width × 6 m diameter	+9 dB
undershot	Germany	7 m diameter	Pulsating noise 2–3.5 Hz
stream water wheel	Germany		

## Data Availability

All the available data are included in the manuscript.
